# High content imaging quantification of multiple in vitro human neurogenesis events after neurotoxin exposure

**DOI:** 10.1186/s40360-016-0107-4

**Published:** 2016-12-01

**Authors:** Xian Wu, Anirban Majumder, Robin Webb, Steven L. Stice

**Affiliations:** 1 Interdisciplinary Toxicology Program, University of Georgia, Athens, GA 30602 USA; 2 Regenerative Bioscience Center, University of Georgia, Athens, GA 30602 USA; 3 ArunA Biomedical, Athens, GA 30602 USA

**Keywords:** Developmental neurotoxicity, Neuron maturation, Neurite outgrowth, Endocrine active compounds, Human neural progenitor

## Abstract

**Background:**

Our objective was to test neural active compounds in a human developmental neurotoxicity (DNT) model that represents neural tube stages of vulnerability. Previously we showed that 14 days in vitro (DIV 14) was sufficient to generate cryopreserved neuronal cells for post thaw neurite recovery assays. However, short exposure and assessment may not detect toxicants that affect an early neurogenesis continuum, from a mitotic human neural progenitor (hNP) cell population through the course of neurite outgrowth in differentiating neurons. Therefore, we continuously exposed differentiating hNP cells from DIV 0 through DIV 14 to known toxicants and endocrine active compounds in order to assess at DIV 14 effects of these compounds in a human DNT maturation model for neurogenesis.

**Methods:**

The Human DNT continuum (DIV 0 to DIV 14) was determined using immunocytochemistry for SOX1+ (proliferating hNP) and HuC/D+ (post mitotic neurons). The cumulative effects of five compounds was observed on neurite outgrowth in (βIII-tubulin+) and (HuC/D+) cells using high content imaging. All data were analyzed using a one-way ANOVA with a significance threshold of *p* < 0.05.

**Results:**

During maturation in vitro, the neural cultures transitioned from uniform hNP cells (DIV 0) to predominantly mature post mitotic neuronal neurons (HuC/D+, 65%; DIV14) but also maintained a smaller population of hNP cells (SOX1+). Using this DNT maturation model system, Bis-1, testosterone, and β-estradiol inhibited neuronal maturation at micromolar levels but were unaffected by acetaminophen. β-estradiol also disrupted neurite extension at 10 μM. Treating cells in this window with Bisphenol A (BPA) significantly inhibited neurite outgrowth and branching in these continuum cultures but only at the highest concentrations tested (10 μM).

**Conclusions:**

Cumulative effects of neurotoxicant exposure during a maturation continuum altered human neurogenesis at lower exposure levels than observed in acute exposure of static cryopreserved neurite recovery neurons cultures. Unlike prior acute studies, β-estradiol was highly toxic when present throughout the continuum and cytotoxicity was manifested starting early in the continuum via a non-estrogen receptor α (ER α) mechanism. Therefore, the effect of neural developmental neurotoxins can and should be determined during the dynamic process of human neural maturation.

**Electronic supplementary material:**

The online version of this article (doi:10.1186/s40360-016-0107-4) contains supplementary material, which is available to authorized users.

## Background

There is overwhelming evidence that environmental factors play a role in the development and progression of a host of central nervous system disorders. Neurotoxins can affect human neural progenitor (hNP) cell to neuron differentiation, survival, proliferation and cellular functions during neurogenesis (such as neurite outgrowth), resulting in profound functional and behavioral deficits in an exposed developing human central nervous system (CNS) [1]. The concept of an embryonic and fetal basis for adult disease has emerged from these findings and has received considerable attention in the scientific community [2, 3]. The extent of damage may be related to not only exposure level, but also exposure duration and developmental stage of exposed neural cells.

Several teratogens are thought to mainly affect early stages of neural maturation occurring during and shortly after neural tube formation [4, 5]. In vivo windows of susceptibility (WOS) were observed when valproic acid (an anticonvulsant that increases the risk of spinal neural tube defects by roughly ten times) was taken early in pregnancy. Valproic acid acts as a histone deacetylase inhibitor and disturbs the balance of protein acetylation versus deacetylation, leading to disruption of key signaling pathways in neurulation during neural tube formation [5]. Retinoic acid (RA) has long been studied as a potent teratogen in rodent systems, with neural tube defects among the malformations observed. Any disturbance in the balance between production and turnover of retinoids can adversely affect developmental events including neural tube closure [5]. Using an in vitro model of early neurogenesis events, human pluripotent stem cell (hPSC) derived neural rosettes responded to retinoic acid exposure with decreased viability and decreased neural rosette formation at a concentration of 2 μM [6]. Disturbance of any of the sequential events of embryonic neurogenesis produces neural tube defects, with the phenotype (e.g. anencephaly, spina bifida) varying depending on the region of neural tube that remains open.

Human PSC-derived neurons can mimic some of the early human neural maturation events, providing in vitro screening opportunities to identify potential developmental neurotoxins [7, 8]. Human NP cells differentiated from hPSC offer a potential cell source for cell based human DNT assays. Human PSC-derived hNP cells, used in this study, proliferate and maintain a multipotent neural state in presence of leukemia inhibitory factor (LIF) and fibroblast growth factor 2 (FGF2) in neural medium [9, 10]. Removal of FGF2 leads to a significant decrease in proliferation within 48 h followed by neuronal differentiation. At fourteen days in vitro (DIV 14), the differentiated cultures show extensive expression of βIII-tubulin (TUJ1), and express active ion channels characteristic of post-mitotic neurons [11]. These differentiated cultures express subunits of glutamatergic, GABAergic, nicotinic, purinergic, and transient potential receptors, and are responsive to neurotransmitters [7, 11]. Therefore, hPSC-derived hNP cells and derivatives mimic early neuronal maturation events in vitro, providing the opportunity to experimentally assess the disruption of neural developmental and proliferative processes.

Neurite outgrowth is an established quantitative endpoint for in vitro monitoring of key cellular events in neuronal differentiation and maturation. Human neuronal cells derived from hNP cell cultures, DIV 14 post removal of FGF2, referred to as hN2 neurons, were used in an in vitro neurite extension/recovery assay for DNT [7]. Compared to a rat primary cortical culture, human hN2 neurons had a lower dynamic range for detecting chemical-induced neurite outgrowth inhibition [7]. Known neurotoxins such as bisindolylmaleimide I (Bis 1) and lithium chloride (LiCl) inhibited neurite outgrowth prior to affecting hN2 neuron viability, inducing cytotoxicity only at higher doses [7, 12]. Past studies, however, only observed effects on mature neurons 2 to 24 h post thaw following acute neurotoxin exposure, providing information on neurite recovery in a mature cell type during a narrow window of development [7]. More informative DNT studies would ideally identify potential human toxicants applied during a continuum of critical early stages of neurogenesis.

Human exposures to endocrine active compounds (EACs) have been implicated in developmental complications [13, 14]. EAC exposure can perturb developmental programming [15] and potentially transgenerational inheritance via changes in the intracellular pathways leading to neural dysfunction [16]. Despite the need, efforts to identify EACs have been limited to only a small subset of the >100,000 synthetic and naturally occurring plant-derived phytoestrogens [17]. This has left many unanswered questions pertaining to the safety of these chemicals and their effects on developmental processes, including neural development. In vitro assays for these outcomes examine genetic changes in cells and are typically accompanied by tests of cytotoxicity and cell proliferation, but they rarely span developmental processes and have not included the neural maturation process.

Human PSC-based DNT assays may provide a relatively rapid test to prioritize which EACs should undergo expensive and time consuming animal DNT studies while also providing further information on potential susceptibility during the continuum of human neurogenesis. To fill this gap, here we examined the effects of three known EAC compounds: β-estradiol, testosterone and Bisphenol A (BPA), on early neural development using a DNT model spanning a characterized DIV 14 neurogenesis period. β-estradiol affected both early stage hNP cell proliferation and neurite outgrowth, effects likely mediated via an estrogen receptor α (ER α) independent mechanism given the absence of ER α expression during this stage of maturation. Testosterone affected neuron densities without significantly affecting neurite outgrowth morphology. BPA effects were less prevalent and only affected neurite branching at the highest dose tested (10 μM) but did not affect the number of neurons. This study combines both neural maturation and cellular function as a cumulative endpoint in the neurogenesis continuum DNT system and can be used for high content and higher throughput screening of larger compound libraries.

## Methods

### Cell culture and differentiation

Cryopreserved hNP cells (hNP1^TM^ 00001) derived from hPSC line WA09 (WiCell 0062, Madison, WI) as previously described [9] were obtained from ArunA Biomedical, Inc. hNP cells were thawed and cultured for 24 h as described by the manufacturer on Matrigel 1:100 (B&D) coated cell culture dishes in proliferation medium comprised of AB2™ basal medium supplemented with ANS™ neural supplement (both from ArunA Biomedical Inc. Athens GA), 2 mM L-glutamine (Gibco), 2 U/ml penicillin (Gibco), 2 μg/ml streptomycin (Gibco), 20 ng/ml bFGF (R&D) and 10 ng/ml leukemia inhibitory factor (LIF) (Millipore, Billerica, MA, USA). After 24hs, hNP cells in dishes were washed with warm phosphate buffered saline (PBS++) and plated on Costar® 96-well cell culture plates at 15,000 cells per well. The 96 well plates were coated with Matrigel 1:100 (B&D) for 20 min (37 °C), rinsed once with warm PBS prior to seeding. These hNP cultures remained in proliferation medium for 24 h, and at the end of this time proliferation medium was exchanged with differentiation medium (proliferation medium lacking bFGF) for 6 h prior to chemical treatment. A 50% differentiation medium change was performed every 2 days. Cells were fixed at different time points (DIV 0, 8, 10, 12, 14, 21, 28) for HuC/D expression quantification and only at DIV 14 for chemical effect staining and quantification. Cell cultures were maintained in a humidified incubator at 37 °C with a 95% air/5% CO2 atmosphere. For experiments determining the effects of test compounds on hNP cells, timing of the addition of compounds to cell cultures is described below.

### Chemical treatment

Test compounds Bisindolylmaleimide I (Bis1), Bisphenol A (BPA), and acetaminophen were purchased from Sigma Aldrich (St Louis, MO), testosterone and β-estradiol were purchased from Steraloids (Newport, RI). All chemicals were prepared as a stock solution (1000×). Bis1 was dissolved in DMSO at concentrations as follows: 0, 0.1, 0.3, 1, 3, 10 mM. BPA and all other chemicals were at concentrations 0, 0.01, 0.1, 1, 10 mM. Selection of concentration ranges was based on previously published work [12, 18–20]. Stock solutions were diluted in differentiation medium at a ratio of 1: 500 and 100 μl mixed medium was added to cell cultures 6 h after cells were incubated in differentiation medium in 96 well culture plates. For second and later medium changes, stock solutions were then diluted in differentiation medium 1:1000 and 100 μl medium was replaced every 2 days. Cells were fixed at the end of DIV 14. Final DMSO concentrations were 0.1% for all treatment wells and corresponding vehicle only control wells.

### Cell proliferation assay for β-estradiol

Human NP cell proliferation was analyzed using CellTiter 96AQueous One Solution Cell Proliferation assay kit (Promega, Madison, WI). This kit contains (3-(4, 5-dimethylthiazol-2-yl)-5-(3-carboxymethoxyphenyl)-2-(4-sulfophenyl)-2H-tetrazolium) (MTS) tetrazolium compound, which is reduced by NADPH produced by dehydrogenase enzymes in metabolically active cells into a colored formazan product. The quantity of formazan product as measured by absorbance at 490 nm is directly proportional to live cells and can be used as a measure of cell proliferation. Cryopreserved hNP cells were thawed cultured for 24 h in culture dishes in proliferation medium, and then hNP cells were seeded on matrigel-coated 96-well plates at 2.5 × 10^4^ cells per well in proliferation medium for 24hs and changed to differentiation medium for 6 h prior to β-estradiol treatment. A set of wells on each plate contained medium and no cells and served as ‘medium only’ background controls. CellTiter 96 AQueous One Solution Reagent was added to each well at end of DIV 3 and DIV 14 β-estradiol treatment, followed by incubation for 2 h at 37 °C in a humidified, 5% CO2 atmosphere. Absorbance at 490 nm was measured for each well using a μQuant Bio-Tek 96-well plate reader. The percentage of cell proliferation was calculated using the following formula: proliferation = 100 × [(experimental − culture medium background)/(medium only control group − culture medium background)].

### Western blotting

Human ESC derived germ-like cells, (GLCs), (provided by Franklin D. West [21]) and IMR90 fibroblast cells (CCL-186™) (ATCC, Manassas, VA) were thawed and plated onto matrigel-coated plates in 20% knockout serum replacement (KSR) medium consisting of Dulbecco’s modified Eagle’s medium/F12 supplemented with 2 mM glutamine, 0.1 mM nonessential amino acids, 50 U/ml penicillin, 50 mg/ml streptomycin (Gibco, Grand Island, NY), 0.1 mM β-mercaptoethanol (Sigma Aldrich, St Louis, MO), and 4 ng/ml bFGF (R&D Systems, Minneapolis, MN) and allowed to acclimatize for 24 h. Cryopreserved hNP cells in proliferation medium and DIV 14 cells in differentiation medium were also cultured for 24hs. After 24 h in culture these cells were used for western blotting and immunocytochemistry.

Cells were lysed in IP lysis buffer complete with Halt protease and phosphatase inhibitor cocktail (both from Pierce, Rockford, IL), on ice for 5 min. Insoluble material was pelleted at 13,000 × g for 10 min per the manufacturer’s protocol, and supernatant collected. Total protein content was determined by bicinchoninic acid assay (Pierce, Rockford, IL), and 7 μg of protein per cell type was separated by SDS-PAGE using 4–12% bis-tris gels (Biorad, Hercules, CA). Proteins were transferred to 0.45 μM nitrocellulose membranes (Biorad, Hercules, CA), and blocked overnight in Li-Cor’s PBS based blocking solution (Li-Cor, Lincoln, NE). Estrogen receptor α was detected using ~ 5 μg of primary (Santa Cruz, Dallas, TX), and Li-Cor’s anti-rabbit IRDye 680 LT secondary, while β-actin was detected using β-actin antibody detected using ~ 1 μg of primary antibody and Li-Cor’s IRDye 800CW conjugated secondary (Li-Cor, Lincoln, NE). Both secondaries were used at the manufacturer’s recommended dilutions, and membranes were imaged on the Odyssey western blot detection system (Li-Cor, Lincoln, NE).

### Immunocytochemistry

For immunocytochemistry cells were fixed with 4% paraformaldehyde - at each time point as described previously [22]. Briefly, 100 μl of a warm (37 °C) solution of 8% paraformaldehyde were added to culture wells containing 100 μl of medium and incubated at room temperature for 20 min [23]. Fixative was then gently aspirated and cells were washed three times with phosphate-buffered saline. Primary antibodies diluted in intracellular blocking solution [24] were then applied as follows: βIII-tubulin 1:300, AB18207 (ABCAM, Cambridge, MA), HuC/D 1:40, A21271 (Invitrogen Corp., Carlsbad, CA), estrogen receptor α 1:50, sc-543 (Santa Cruz, Dallas, TX), SOX1 1:300, AF3369 (R&D, Minneapolis, MN) for 2 h at RT. The entire antibody panel was used to characterize differentiation and phenotype of the hNP and DIV 14 neural cells, while βIII-tubulin was specifically used to label cell bodies and neurites for high-content image analysis. Following incubation in primary antibodies, cells were washed three times with high salt buffer and incubated with a 1:400 dilution of DyLight® 594-conjugated donkey anti-mouse IgG or DyLight® 488-conjugated donkey anti-rabbit IgG secondary antibody in high salt buffer for 1 h at room temperature, protected from light. Cells were then incubated in 0.1% Hoechst 33342 dye in high salt buffer for 20 min, then in PBS washed 3 times with high salt buffer, and stored in phosphate-buffered saline (PBS) at 4 °C prior to image acquisition and analysis [7].

### Image acquisition and analysis

Cellomics ArrayScan VTI HCS reader high-content imaging system (ThermoFisher Scientific, Waltham, MA) was used for automated image acquisition and morphometric analyses as previously described for use on hN2 cells [7]. Image analysis was performed using the vHCS Scan software package with a manually optimized version of the Cellomics Neural Profiling Bioapplication for neurite outgrowth analysis. Target Activation Bioapplication was used for marker protein expression analysis. Image analysis algorithm optimization, including nucleus validation, cell body masking and validation, and neurite tracing parameters, was performed a priori using five representative images from cultures with differentiated neural cells. Output from high content image analysis included total cell count (% viable nuclei per well) and measurements of neurite outgrowth (neurites per neuron, neurite length per neuron, and branch points per neuron). Anti-human neuronal protein HuC/HuD recognizes multiple neuronal proteins of the Elav family; HuC, HuD and Hel-N1and labels neuronal cells at the time that neurons leave the mitotic cycle [25–27]. HuC/D protein positive expression was analyzed in Target Activation Bioapplication. Briefly, nuclei were first identified in channel 1 as bright objects on a dark background (Fig. 2a, b). Nuclei with size and intensity values outside of the ranges determined a priori for viable cells were identified in the channel 1 image and rejected from further analysis [7]. Briefly, to determine live cells and exclude dead cells, Cellomics Neural Profiling Bioapplication was used to distinguish live cell nuclear based on average intensity and area from dead cells. The bioapplication calculated live cell nucleus hoechst staining average intensity and area. To be considered a live cell, the nucleus had a hoechst staining intensity below 1000 and nuclear area above 65. A nucleus with intensity score above 1000 or nuclear area below 65 were considered dead cell and excluded for analysis (W Mundy, personal communications). Spatial coordinates from the channel 1 image were then superimposed on the matching channel 2 image. Protein expression in channel 2 were then cast based on positional data from channel 1 nuclei and a set of user-defined geometric and signal intensity-based parameters (Fig. 2c, d. orange and red traces). Positive objects based on intensity value were then selected (Fig. 2c, d. red traces) and invalid objects excluded (Fig. 2c, d. orange traces). In neurite outgrowth analysis, Hu C/D staining was quantified in channel 1 while βIII-tubulin staining was quantified in channel 2 based on Hu C/D positional data from channel 1 (Fig. 2h–i). Only cells that were Hu C/D+ and βIII-tubulin + were analyzed for neurite outgrowth. Data were collected on a cell-by-cell basis, and values were averaged to obtain population means within each well. These well level data were treated as the statistical unit for analysis of neurite outgrowth. At × 20 magnification, the Cellomics ArrayScan VTI can sample 81 individual fields within each well. In this study, 35 fields were sampled within each well for cell characterization.

### Statistics

Cell characterization experiments were performed twice using independent cultures with *n* = 4–6 wells per condition per culture. For concentration-response experiments, total cell count, HuC/D positive cell number (neuron density), neurite outgrowth data were normalized within experiment to corresponding control wells prior to statistical analysis. For each concentration-response examined, experiments were repeated two to three times using independent cultures as described. In cell proliferation assay, experimental values are a composite of six technical (on same plate) and three biological (different plates) replicates. All data analyzed for cell characterization were using a one-way ANOVA with a significance threshold of *p* < 0.05. This was followed by a Tukey’s test to determine if different time point means were significantly different from corresponding control means. All concentration-response experiments were analyzed using one-way ANOVA with a significance threshold of *p* < 0.05 followed by a Tukey’s test. Mean values ± standard deviations for all measurements are provided throughout the text. Statistical analyses were performed using Graphpad Prism1 v5.

## Results

### Quantification of neural progenitor cell differentiation using high content analysis

**Fig. 1:**
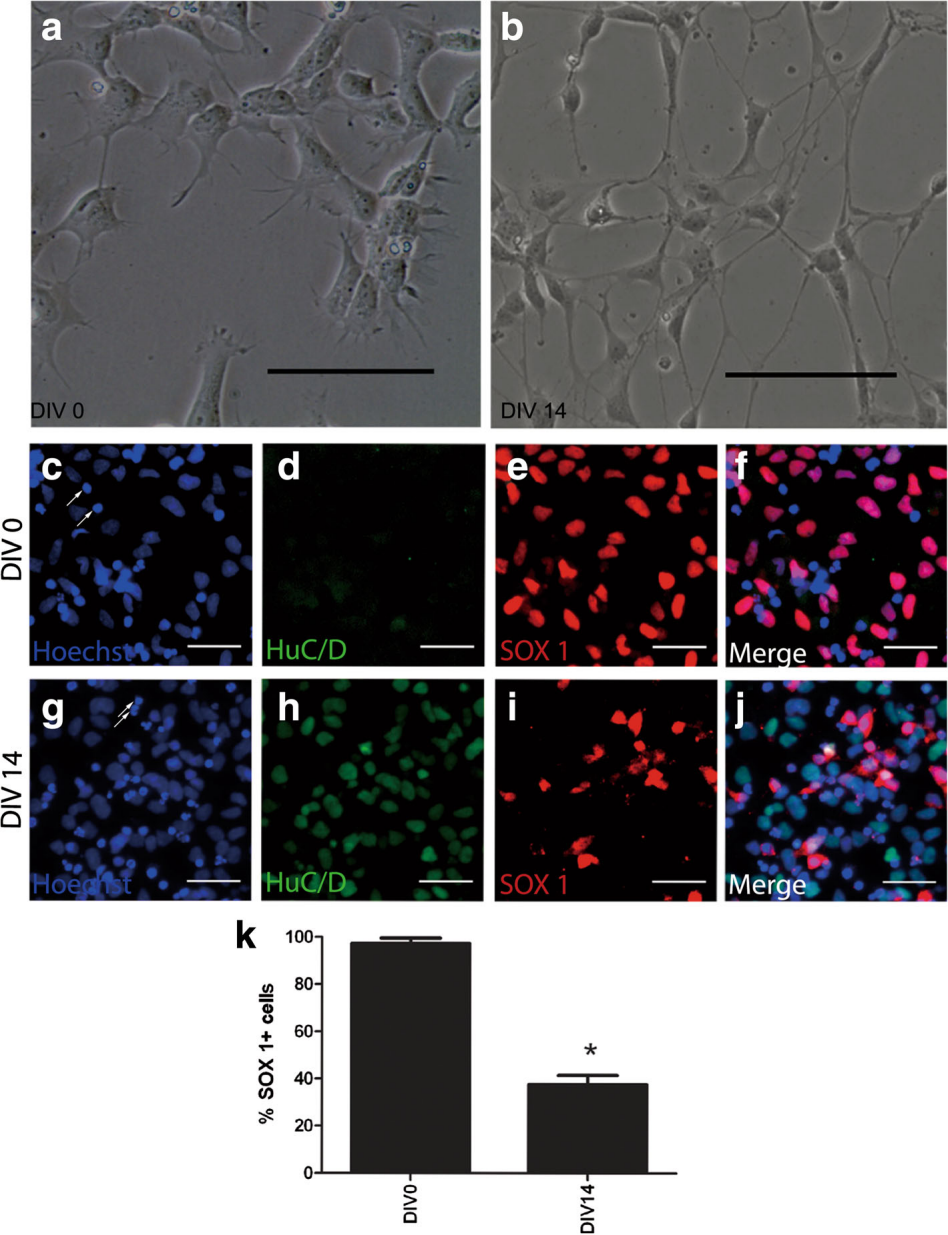
DIV 0 and DIV 14 neural cell morphology and SOX 1 expression quantification. hNP cells were seeded onto 96 well plates at a density of 15,000 cells/well, differentiating hNP cultures were fixed at end of DIV 14 for analysis following immunocytochemistry for HuC/D, SOX 1 and nuclear staining. SOX 1+ cells were then imaged and quantified by Cellomics ArrayScan VTI HCS reader high-content imaging system. **a**, **b**: Phase contrast images of neural progenitor (DIV 0) and neuron (DIV 14). Scale bars = 100 μm. **c**, **g**: DIV 0 and DIV 14 cells hoechst 33342 staining. **d**, **h**: DIV 0 and DIV 14 cells HuC/D staining. **e**, **i**: DIV 0 and DIV 14 cells SOX 1 staining. **f**, **j**: DIV 0 and DIV 14 cells Pseudo colored images. Arrows indicate invalid cells and excluded from quantification. Scale bars = 50 μm. **k**: The quantification of SOX 1 in DIV0 and DIV14 differentiation. Values are the means ± SD. *significantly difference between group (*P* < 0.05)

**Fig. 2:**
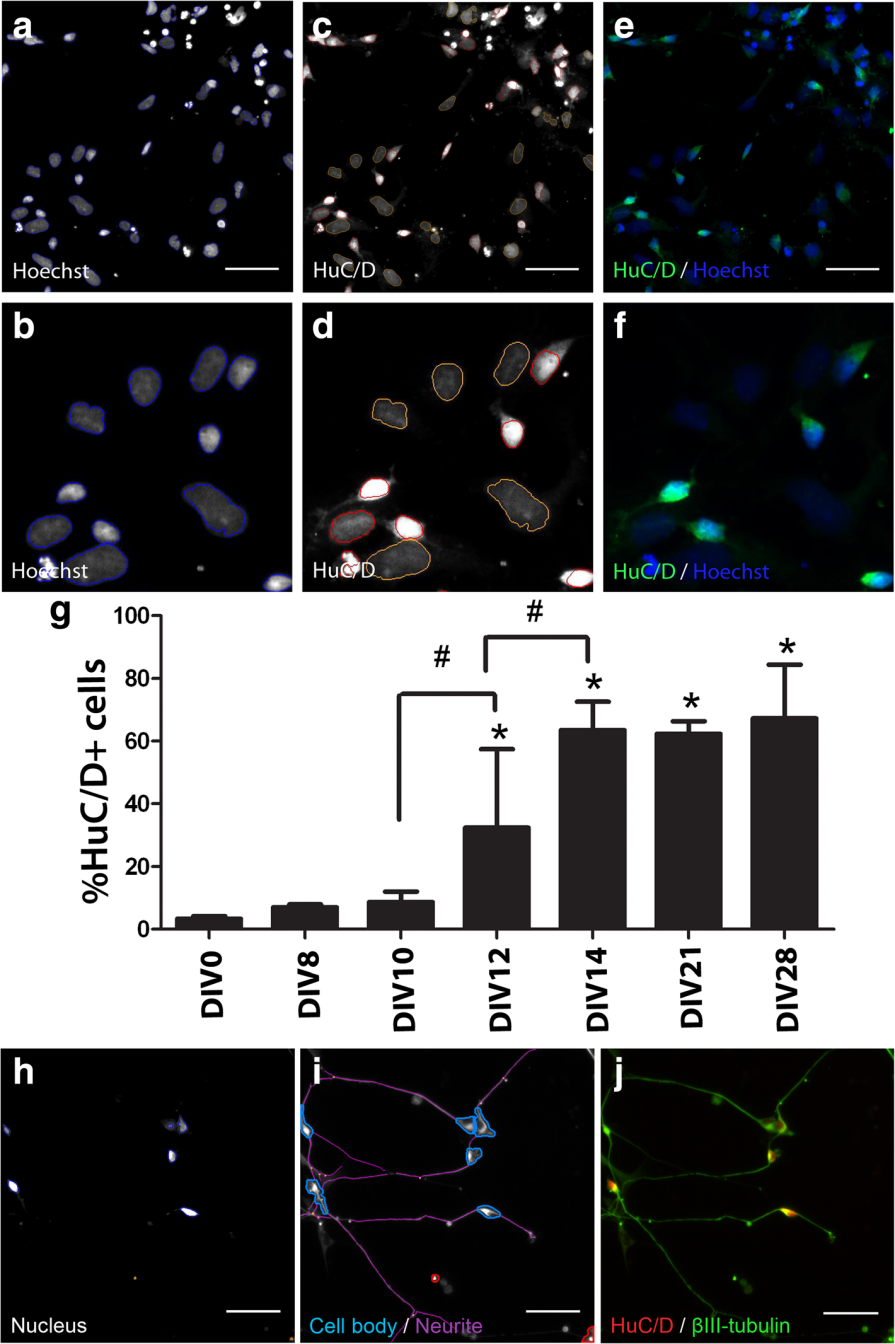
Automated measurement of HuC/D expression and quantification of neurite outgrowth during neural differentiation. hNP cells were seeded onto 96 well plates at a density of 15,000 cells/well, differentiating hNP cultures were fixed at different time points for analysis following immunocytochemistry for HuC/D, βIII-tubulin antibody and nuclear staining. Cells were then imaged and quantified by Cellomics ArrayScan V^TI^ HCS reader high-content imaging system. **a**–**f** and **h**–**j** represent cells at DIV 14. A, B (channel 1): Nuclei stained with Hoechst 33342, live cell nuclei (*blue trace*). **c**, **d** (channel 2): HuC/D+ cells stained (*red trace*), rejected cells stained (*yellow trace*). **e**, **f**: Pseudocolored composite image combining channels 1 and 2. **b**, **d**, **f** are magnified images to illustrate tracing in panel **a**–**c**. Scale bars = 50 μm. **g**: HuC/D+ expression throughout differentiation of neuronal cells. *Concentration is significantly different from control group (*P* < 0.05, one-way ANOVA), ^#^Concentration is significantly different between groups (*P* < 0.05, one-way ANOVA). ***h*** (Channel 1): Nuclei identification. *Blue* trace = accepted, *Yellow* trace = rejected. I (Channel 2): Cell body masks based on β_III_-tubulin and HuC/D expression; *Blue* trace = accepted cell, *Red* trace = rejected cell, *Purple* line = neurite, *Yellow* dot = branch point. Cells marked as rejected are not included calculating neurites per neuron or neurite length per neuron. Neurites emerging from accepted cell bodies are traced (*purple lines*) and quantified. **j**: Pseudo colored images from **c** and **d** merged. Scale bars = 50 μm

Distinct hNP and post mitotic neuronal morphologies were evident at DIV 0 and DIV 14 (Fig. 1a–b, representative images). SOX1 is expressed in hNP cells but not in mature cells [28, 29]. SOX1 positive cells were evident in DIV 0 and represented nearly 100% of the culture. The SOX 1 positive cells decreased to only 37.5% at DIV 14 (Fig. 1c–k); There was no observed co expression of both SOX 1 and Hu C/D (Fig. 1j), whereas HuC/D+ post mitotic neurons were negligible at DIV 0 but was at 63.5% of the population at DIV14 (Fig. 2g). Therefore, hNP cells and post mitotic neurons composed nearly 100% of total live cells quantified by hoechst staining during the neurogenesis continuum. To further understand the transition from mitotic hNP cells to post mitotic neurons in the neuronal maturation continuum, expression of neuronal marker HuC/D was determined continuously at regular intervals from DIV 0 to DIV 28 (Fig. 2g) using a high content imaging format. HuC/D positive cells increased during the first 14 DIV (Fig. 2g). Only 3.4% ± 0.8% of the hNP cells population (DIV 0) expressed HuC/D compared to 63.5% ± 8.5% at DIV 14 and the percentage of HuC/D positive neuronal cells did not significantly increase further after DIV 14, with 67.3% ± 13.9% expressing HuC/D at DIV 28 (Fig. 2g). Thus, HuC/D expression approached a plateau around DIV 14 and was constant for the additional 14 days of differentiation, presenting DIV 0–14 as a window from a proliferative to a largely post mitotic stage. Co-expression of HuC/D and βIII-tubulin specifically labeled cell bodies and neurites, enabling quantification of neurogenesis at DIV 14. HuC/D was present in the nucleus and βIII-tubulin expression was evident in both axons and dendrites of neural cells providing an accurate measure of neurite outgrowth (Fig. 2h–j).

### Effect of test compounds during neurogenesis

The early developmental window described above was used to test a four-point concentration series of one known neurotoxin and protein kinase C (PKC) inhibitor Bis1, an assumed non-neurotoxic drug, acetaminophen, two known endocrine active compounds: testosterone and β-estradiol, and the putative EAC, BPA. Concentration ranges were based on previous research as described in the literature (Table 1) [12, 18–20]. All parameters including neuron density and neurite outgrowth were normalized to percentages of non-treated cells. At the end of DIV 14, neurite outgrowth measurements were as follows: neurite length per neuron: 52.8 ± 6.7 μm; neurites per neuron: 1.3 ± 0.1; branch points per neuron: 0.7 ± 0.1.

**Table 1:** Chemicals with evidence of neurite outgrowth inhibition or enhancement

Compound	Study	Cell type	Effect
Bis 1	Radio et al.	CGC	↓ in neurite outgrowth
β-Estradiol	Rozovsky et al.	cortical neuron	↑ in neurite outgrowth
BPA	Seki et al.	PC 12	↓ in neurite extension
Testosterone	Zhang et al.	Cerebral cortex neuron	↓ Neurite length Neuronal differentiation
Acetaminophen	Radio et al.	PC12, CGC	Not reported in literature

**Fig. 3:**
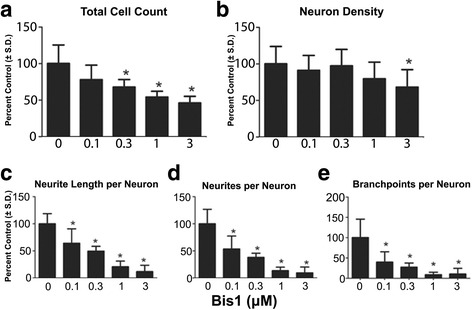
Effects of long term Bis1 exposure on neural differentiation and neurite extension. Cells were cultured on 96 well plates and continuously exposed to a range of doses of Bis1 through DIV 14. Cultures were then analyzed by immunocytochemistry staining for HuC/D along with β_III_-tubulin followed by automated image acquisition and processing. **a**: Quantification of total cell count (total live cells using Hoechst 33342 positive nuclei). **b**: Quantification of neuron density (HuC/D+). Neuron density was measured as an indicator of cell health. **c**, **d**, **e**: Neurites per neuron, neurite length per neuron, branch points per neuron were also measured. All data are presented as % change from untreated control wells. Total live cell count, neuron density and neurite outgrowth data are from 2 separate experiments using independent cultures (*n* = 11 wells total). *Concentration is significant different from control group (*P* < 0.05, one-way ANOVA)

**Fig. 4:**
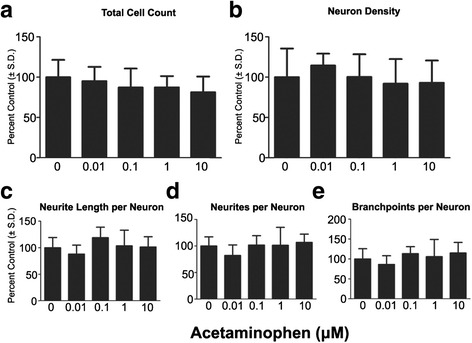
Effects of long-term acetaminophen exposure on neural differentiation and neurite extension. Cells were cultured on 96 well plates and continuously exposed to a range of doses of Bis1 through DIV 14. **a**: Quantification of total cell count (total live cells using Hoechst 33342 positive nuclei). **b**: Quantification of neuron density (HuC/D+). Neuron density was measured as an indicator of cell health. **c**, **d**, **e**: Neurites per neuron, neurite length per neuron, branch points per neuron were also measured. All data are presented as % change from untreated control wells. Total live cell count, neuron density and neurite outgrowth data are from 2 separate experiments using independent cultures (*n* = 12 wells total). *Concentration is significant different from control group (*P* < 0.05, one-way ANOVA)

Following continuous exposure to test compounds up to DIV 14, Bis1 reduced total cell density in culture at all concentrations tested, while only the highest concentration induced a decrease in a differentiated HuC/D+ cell population (3 μM; Fig. 3a, b). In contrast, concentration dependent decreases in neurite outgrowth parameters were observed following exposure to Bis1 (0.1 to 3 μM). The average number of neurites, neurite total length, and branch points per neuron all decreased in a concentration dependent manner (Fig. 3c, d, e). These data demonstrated a specific inhibition of neurite outgrowth ranging from 0.1 to 3 μM Bis1. Previously, Bis1 has been used on cryopreserved DIV 14 pre-differentiated neurons for neurite outgrowth/recovery assays [7] and in agreement, 100-fold higher level of Bis1 exposure is required to induce significant effects with a 24 exposure (Additional file 1: Figure S1). Acetaminophen, the non-toxic control, had no effect on either neuron density or neurite outgrowth at doses ranging from 0.01 to 10 μM (Fig. 4a-e).

**Fig. 5:**
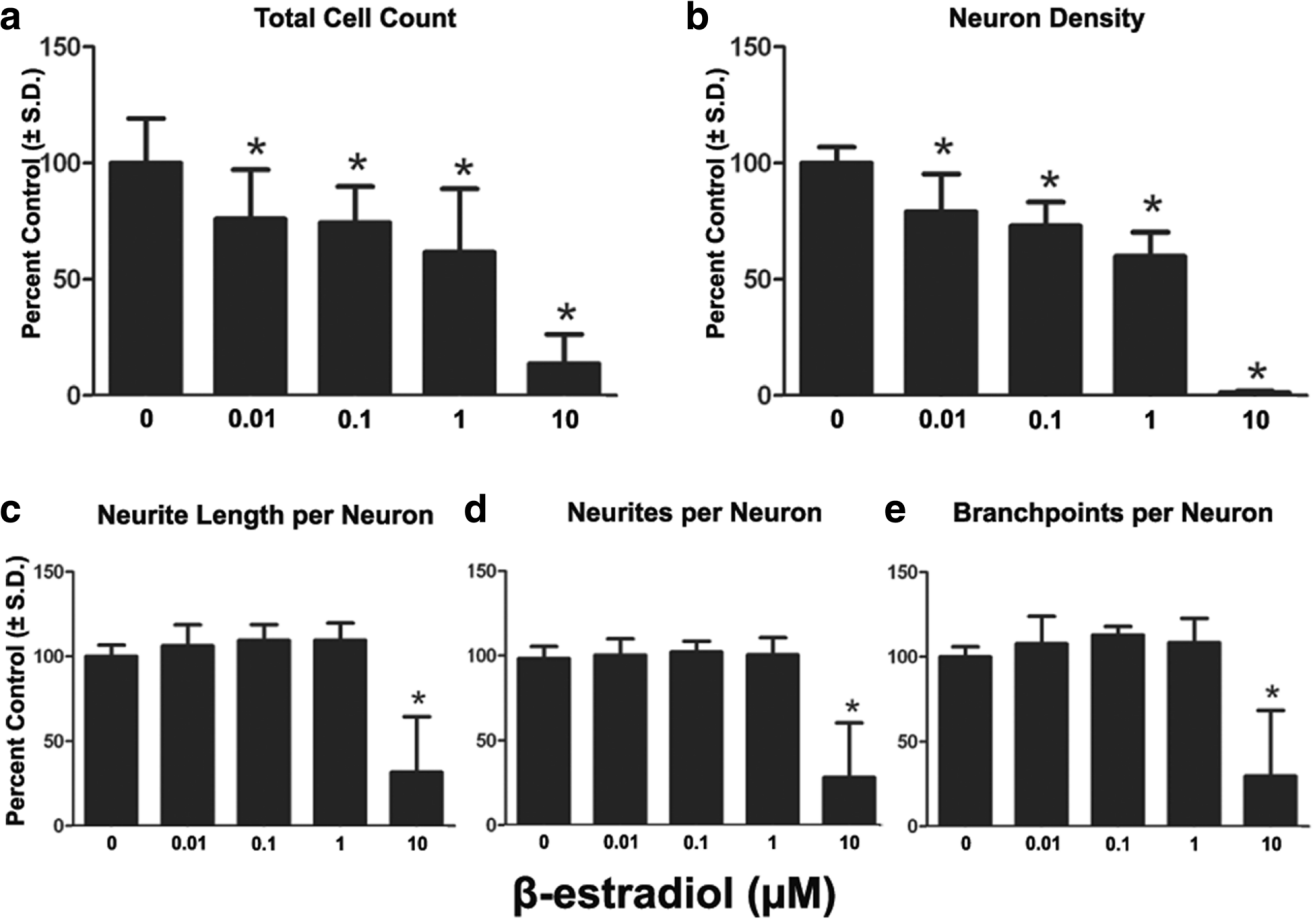
Effects of long-term β-estradiol exposure on neural differentiation and neurite extension. Cells were cultured on 96 well plates and continuously exposed to a range of doses of Bis1 through DIV 14. **a**: Quantification of total cell count (total live cells using Hoechst 33342 positive nuclei). **b**: Quantification of neuron density (HuC/D+). Neuron density was measured as an indicator of cell health. **c**, **d**, **e**: Neurites per neuron, neurite length per neuron, branch points per neuron were also measured. All data are presented as % change from untreated control wells. Total live cell count, neuron density and neurite outgrowth data are from 2 separate experiments using independent cultures (*n* = 11–12 wells total). *Concentration is significant different from control group (*P* < 0.05, one-way ANOVA)

We then tested increasing doses of three EACs in this DNT continuum. β-estradiol treatment led to a significant reduction in total cell count at 0.01–10 μM as measured in Fig. 5a. Additionally significant concentration dependent decreases in HuC/D positive cells (neuron density) were observed following exposure to β-estradiol (Fig. 5b) from 0.01 to 10 μM; a significant decrease in neurite outgrowth was observed only at 10 μM. The number of neurites per neuron decreased by 70.1%. Neurite length per neuron and branch points per neuron were significantly inhibited at 68.3% and 70.3% respectively (Fig. 5c, d, e).

**Fig. 6:**
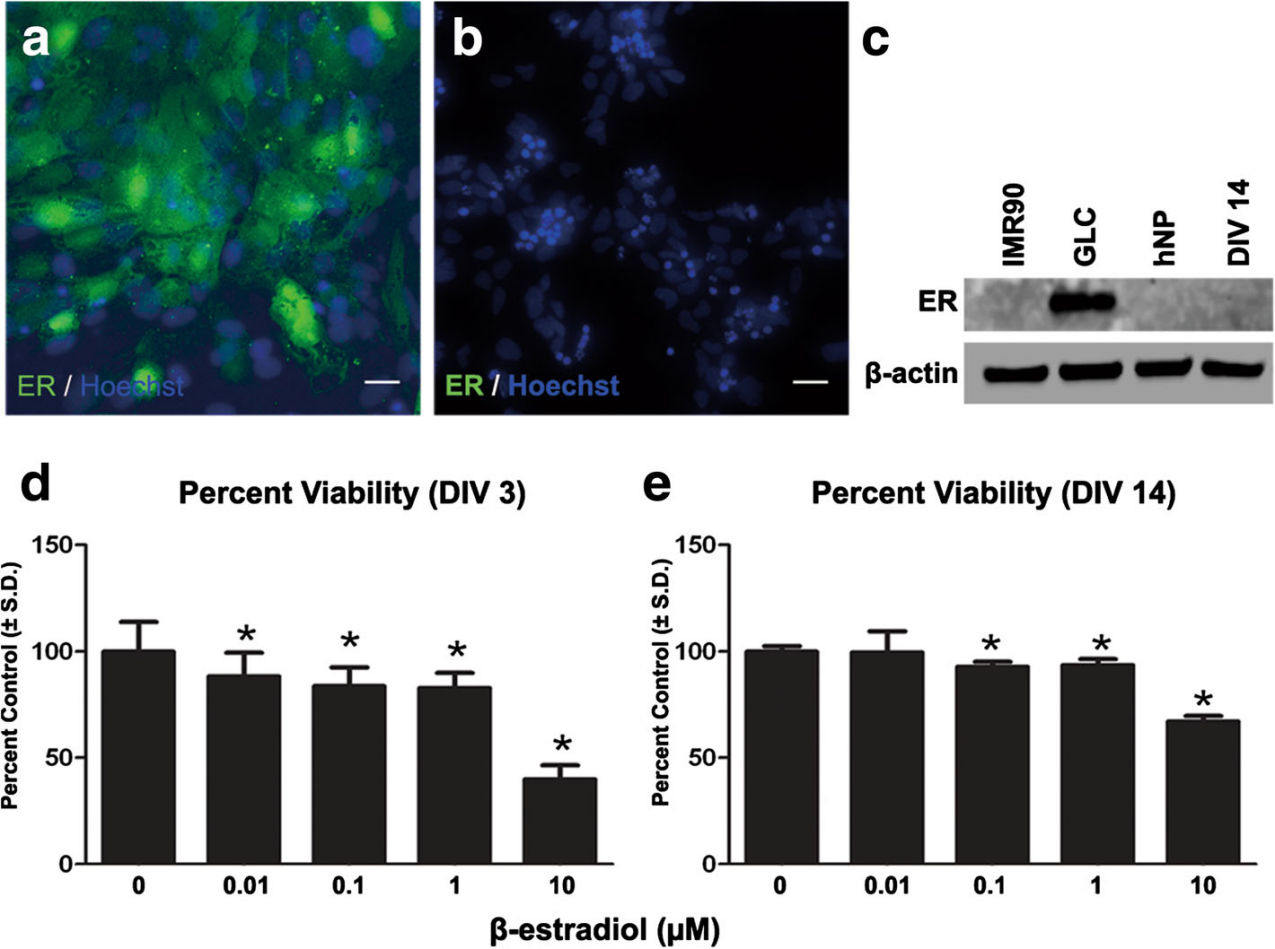
Estrogen receptor α protein expression and effect of β-estradiol on neural progenitor cells proliferation. Germ like cells, hNP cells, DIV 14 neuronal cells were seeded in 8 well slide for 24 h at density of 30,000 cells per well and fixed for estrogen receptor α protein immunostaining. Germ like cells, hNP cells and DIV 14 neuronal cells were also seeded in 60 mm dish and collected at 90% confluent density for western blot experiment. In proliferation assay, hNP cells were seeded onto 96 well plates at a density of 25,000 cells/well, medium was changed with β-estradiol every other day until end of DIV 3 and DIV 14. All data are from 3 separate experiments using independent cultures (*n* = 18 wells total). *Concentration is significant different from control group (*P* < 0.05, one-way ANOVA). **a**: Germ like cell estrogen receptor α staining; **b**: hNP cell estrogen receptor α staining **c**: Western blot analysis of IMR90, GLCs, hNP and DIV 14 neuron estrogen receptor α expression. β-actin was used for normalizing the loading of samples. **d**: DIV 3 cell proliferation assy. **d**: DIV 14 cell proliferation assy. Scale bars = 50 μm

The β-estradiol low dose cytotoxic effects in the high content assay were investigated further given prior studies suggesting β-estradiol can act via receptor mediated or receptor independent mechanisms [30] (Fig. 5a, b). Estrogen receptor α (ER α) was involved in neural proliferation and differentiation in non-human neural cell lines [31, 32]. The hNP cells were negative for ER expression when compared to known ER positive human PSC-derived cell germ-like cell line (Fig. 6a, b) [33]. Additionally, the absence of ER expression in both hNP cells and DIV 14 neural cells was confirmed by western blot (Fig. 6c).

Previously β-estradiol has been shown to influence (either increase or decrease) neural progenitor cell proliferation and neurite extension [31, 34]. β-estradiol decreased hNP cell viability or proliferation early (DIV 3) in neurogenesis (0.1–10 μM dose range); (Fig. 6d). At DIV 14 of continuous exposure to β-estradiol during differentiation, this cytotoxicity assay confirmed cumulative effects in the 0.01–10 μM dose range. (Fig. 6e). These results suggest the effect of β-estradiol is not mediated via an ER mechanism and confirm the cytotoxicity is not cell type specific given both decreased hNP proliferation and viability (DIV 3) and the number of total of differentiated cells in DIV 14 cell culture.

**Fig. 7:**
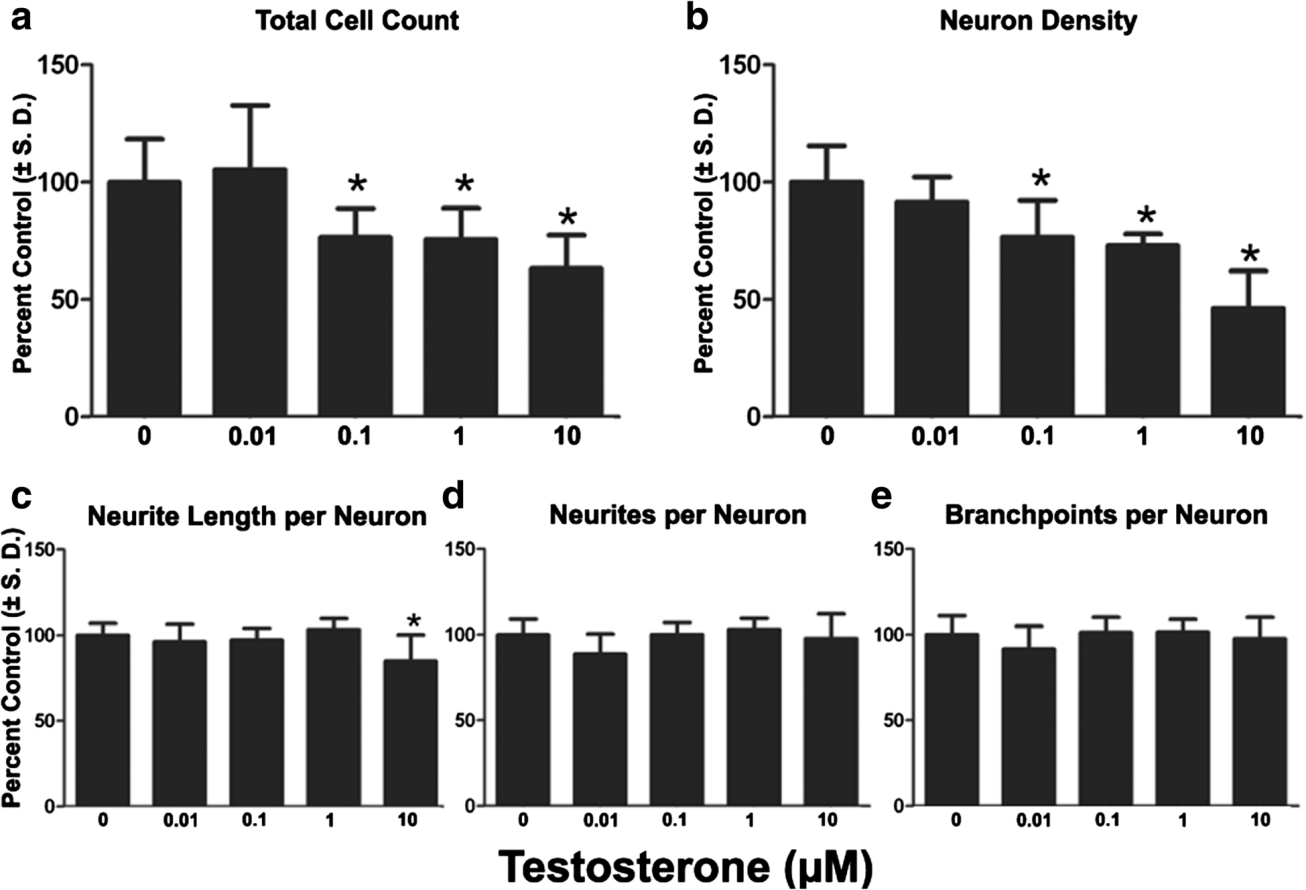
Effects of long-term testosterone exposure on neural differentiation and neurite extension. Cells were cultured on 96 well plates and continuously exposed to a range of doses of Bis1 through DIV 14. **a**: Quantification of total cell count (total live cells using Hoechst 33342 positive nuclei). **b**: Quantification of neuron density (HuC/D+). Neuron density was measured as an indicator of cell health. **c**, **d**, **e**: Neurites per neuron, neurite length per neuron, branch points per neuron were also measured. All data are presented as % change from untreated control wells. Total live cell count, neuron density and neurite outgrowth data are from 2 separate experiments using independent cultures (*n* = 12 wells total). *Concentration is significant different from control group (*P* < 0.05, one-way ANOVA)

Exposure to testosterone affected both total cell count and neuron density. At 0.1, 1 and 10 uM, cell numbers were reduced and neuron densities were significantly decreased compared to control group (Fig. 7a, b). There were thus two concentrations, 0.1 and 1 μM, which affected neuron density without concurrent effect on neurite outgrowth (Fig. 7c, d, e). At 10 μM, neurites per neuron was significantly decreased by 15.0% from control group, while total neurite length nor branch points per neuron were affected over the concentration range tested.

**Fig. 8:**
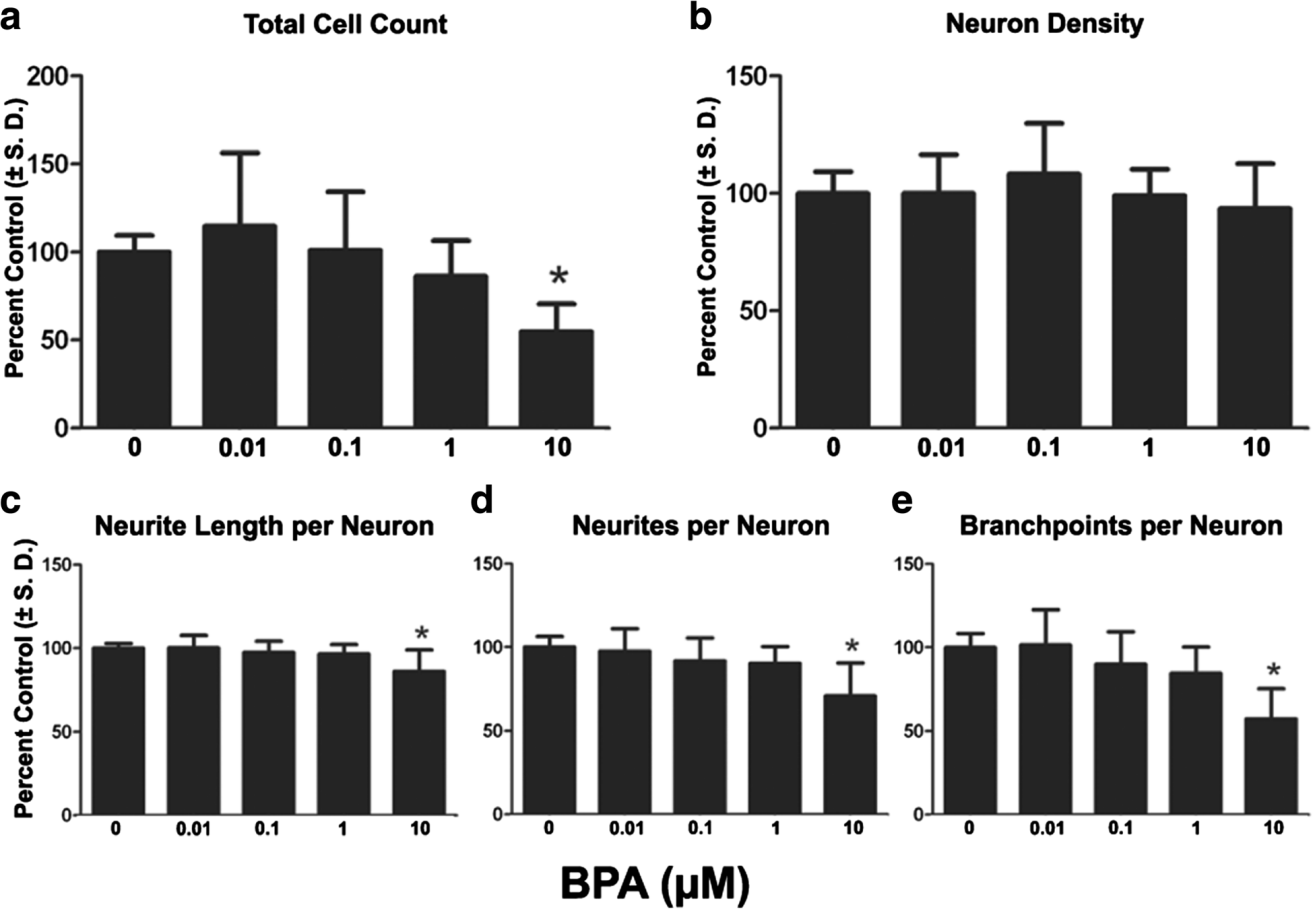
Effects of long-term BPA exposure on neural differentiation and neurite extension. Cells were cultured on 96 well plates and continuously exposed to a range of doses of Bis1 through DIV 14. **a**: Quantification of total cell count (total live cells using Hoechst 33342 positive nuclei). **b**: Quantification of neuron density (HuC/D+). Neuron density was measured as an indicator of cell health. **c**, **d**, **e**: Neurites per neuron, neurite length per neuron, branch points per neuron were also measured. All data are presented as % change from untreated control wells. Total live cell count, neuron density and neurite outgrowth data are from 3 separate experiments using independent cultures (*n* = 18 wells total). *Concentration is significant different from control group (*P* < 0.05, one-way ANOVA)

BPA exposure decreased total cell count by 45.2% at the highest dose tested (10 μM) while lower concentrations did not induce toxicity. Even though total cell count was substantially reduced, neuron density did not change with BPA treatment at any concentration tested (Fig. 8a, b). Importantly, even though neuron density did not change at any concentration tested, all three neurite outgrowth endpoints were reduced at highest concentration. Following exposure to 10 μM BPA, significant decreases of 29.0%, 13.9%, and 42.8% were observed for neurite length per neuron, neurites per neuron, and branch points per neuron respectively (Fig. 8c, d, e). Collectively, all data from the chemical exposures indicate that EACs affect neuron maturation and neurite outgrowth differently, but induce changes within this window, supporting use of the continuum model over single endpoint systems.

## Discussion

The current study establishes an in vitro developmental window in a hPSC-based early neurogenesis system that specifically addresses toxin susceptibility during a continuum of early maturation that is observed in vivo. In the neural tube the neuroepithelium proliferates and then post mitotic neurons migrate basally to form the neocortex [35]. This is a dynamic process, including but not limited to continued cell proliferation and apoptotic events. Human PSC-based neural development has been shown to represent aspects of human neural development. In vitro derived neural rosettes have been used to model the early neural tube [36–38], polarized post mitotic neurons migrate apically as they do during embryonic development. hNP cells are derived from neural rosette structures which under repeated passage retain their differentiation potential but become non-rosette hNP cells which are Nestin + and SOX2+ and GFAP- in bFGF free media [9]. Upon removal of bFGF from the culture medium, hNP cell population starts to differentiate as a whole, with arrested cell cycle of hNP cell population arrested in by 96 h, followed by an increase in pan neuronal marker TUJ 1 expression. The differentiating population also exhibit cell death during this process [11]. However, these are mixed cultures containing mostly neurons, positive for Hu C/D and TUJ 1, and initially hNP cells, with a minimal, if any astrocytes or other non-neuronal cells [9]. Here we used the early developmental continuum and assay that tested the cumulative effects of neurotoxin and EAC exposure throughout an early maturation process in a high content analysis system, which allowed for measurements of total live cell and exclusive measurements on the HuC/D+ and βIII tubulin + population.

In experiments where we assayed differentiating hNP cells for expression of the neuronal marker HuC/D [39], the observed increase in HuC/D expressing cells through DIV 14, which then plateaued through DIV 28 (the furthest time point tested) suggests we are modeling a population shift, from a proliferating population to a primarily post-mitotic one. However, other non HuC/D positive, SOX 1+ cell were also present by DIV 14, suggesting a mixed culture, prompting the need and use of high content imaging to specifically measure neural outgrowth in cells that coexpressed HuC/D and βIII-tubulin. Compared to other neural differentiation models [40, 41], this is the first in vitro model measuring both maturation and neurite outgrowth during neural differentiation based specifically on the HuC/D positive cells, and in turn distinguishing a compound’s effect on total live cells vs only the HuC/D+ neurons (density and outgrowth parameters).

At the end of DIV0 to 14, we observed extensive neurite outgrowth in control non treated cultures, providing a robust assay endpoint after toxin exposure during a developmental continuum; from neurite budding to neurite outgrowth. Here we encompass an in vitro representation of neurite outgrowth. We observed neurite extension starting approximately at DIV 8 and an extensive neurite outgrowth was formed by DIV14, which allows toxin treatment to occur concurrently with neurite extension. This is in contrast to the use of cryopreserved DIV 14 neuronal cultures that lose neurite morphology during cryopreservation and essentially recover and reestablish their neurites during toxin treatment post-thaw [7].

Bis1 has been studied extensively for effects on neurite recovery in vitro, using rodent and human cells [42]. Bis1 is a competitive inhibitor for the ATP binding site of PKC. PKC can affect neurite outgrowth directly, modulating cytoskeletal protein phosphorylation, or through the MAPK signaling pathway which is active in our differentiating cultures [8, 43]. In a previous study, where cryopreserved hN2™ neurons (14 DIV) were exposed to Bis1 for a short (24 h) period post thaw, significant inhibition of neurite outgrowth was observed [7]. We repeated the acute Bis1 exposure to cryopreserved DIV 14 culture, to confirm and compare with the present study (Additional file 1: Figure S1). The least effective concentration being 10 μM for both neurites per neuron and neurite length per neuron (28.8% and 36.7%), with no cytotoxicity observed at this concentration ([7] and Additional file 1: Figure S1). Bis1 did not inhibit neuron density at 10uM in 24 h acute study ([7] and Additional file 1: Figure S1). However, no live cells remained after 14 days neural maturation in 10 μM Bis1 (Additional file 1: Figure S1) and prompted the use of a lower Bis1 dose range (0.1 to 3 μM) to measure the cumulative effects during maturation. A significant reduction in neurites per neuron and neurite length per neuron as well as branch points per neuron was observed with a dose of 0.1 μM, a reduction of 2 logs during the 14 DIV, suggesting significantly more sensitivity in a model where the toxicant exposure occurs during neural differentiation. No effect of Bis1 on neuron density was observed at this concentration, showing endpoint specificity on neurite outgrowth. To ensure that the increased sensitivity is not at the expense of specificity, we also tested acetaminophen, a drug with no reported neurotoxicity in vivo, except with acute overdose. No significant effect was observed at any dose up to 10 μM, the highest tested. Together, extended exposure to Bis1 throughout maturation, spanning from hNP cells to HuC/D+ cells, generated higher levels of toxicity at 100 fold lower dose concentration, compared to the acute exposure of cryopreserved neurons (Fig. 3). These Bis 1 data suggest that DNT effects are more significant if the exposure is throughout the continuum of maturation. A study with a larger library of compounds with different exposure periods can now be conducted to specifically understand which stages in the continuum of neural maturation are affected by test compounds.

We tested the effects of known EACs in this DNT maturation model. EACs are found in many environmental agents including pesticides, industrial chemicals and some plasticizers and surfactants, and are thus of interest to regulatory and public health agencies [44]. Two known EACs: Testosterone and β-estradiol as well as putative EAC, BPA, were tested. Although it is difficult to compare directly with our in vitro studies, it is important to keep in mind what levels of EAC are present in humans. Serum levels of estradiol varies from 10 to 50 pg/mL in males, 30 to 400 pg/mL in premenopausal females and 0 to 30 pg/mL postmenopausal. Upon human chorionic gonadotropin administration in assisted reproduction, serum estradiol levels were 10,000 pmol/L which equals the concentration of 0.01 μM which we found in this study to be neurotoxic in our DNT model [45].

Following continuous exposure to β-estradiol, all three neurite outgrowth parameters were affected at 10 μM, the highest dose tested. However, both the number of total cells and neurons were significantly reduced at lower concentrations starting from 0.01 μM. Estrogen effects on neurons have been studied extensively. Estrogen induced neurite outgrowth in rat basal forebrain cholinergic neurons in vitro, [46] and β-estradiol had a neuroprotective effect [47]. 10 nM estradiol increased the proliferation of rat primary neural stem cells and increased the ratio of neurons to glia cells in embryonic neural stem cells. These effects were induced via estrogen alpha receptor. Pre-incubation with estradiol provided significant neuroprotection against glutamate-induced neurotoxicity with ED50 at 50 μM for dopaminergic neurons, and this effect was not blocked by estrogen receptor antagonist. This study indicated that at high concentration neuroprotective effect of estradiol was not mediated through estrogen receptors [48]. Both neural progenitors and differentiated neurons that represent the two temporal boundaries for our treatment window were tested for ER protein expression. The absence of ER protein expression in both neural stages suggests that the effects of β-estradiol we observe occurs through ER independent mechanisms. Such ER independent mechanisms can affect cytoplasmic microtubule fibers at interphase, and affect cell growth [49]. We show that β-estradiol, when applied during the first 3 days of our DIV 14 developmental window, increased cytotoxicity in hNP cells, but may have had a less pronounced effect on maturing neurons, given the lower cytotoxicity levels at DIV 14 (Fig. 6).

BPA significantly reduced neurite outgrowth, including neurite length per neuron, neurites per neuron and branch points per neuron at 10 μM, the highest dose tested, without reduction in the number of neurons (Fig. 8). This shows endpoint specificity, and suggests higher sensitivity than a cytotoxicity based endpoint for detecting effects of BPA. However, we did observe a reduction in the overall number of cells in cultures exposed at this dose, suggesting a decrease in the proportion of non-neuronal (HuC/D negative or immature) cells by DIV 14 due to continuous BPA exposure. Interestingly, the 10 μM dose point that elicited an effect is significantly higher than the effective dose of 10 ng/ml observed previously with PC12 cells [18] in spite of the long term treatment performed here, suggesting a cell line and/or species specific difference in sensitivity with human cells being less responsive to the effects of BPA.

With long-term exposure to testosterone, the highest dose tested reduced the number of neurites per neuron, but had no effect on other parameters such as neurite length per neuron or branch points per neuron, suggesting non-endpoint specific effects since neurite outgrowth was affected only at 10 μM. However, a significant reduction in both total cells and neurons was observed at lower doses, consistent with previous reports that testosterone in the micromolar range initiates apoptosis and decreases neural cell viability [41].

In summary, we provide evidence that an hPSC-derived neurogenesis continuum DNT model can be used to test EACs and other compounds, providing a multifaceted and potentially more complex understanding of neurotoxin effects during early human neuronal development. Here cell cultures spanning from proliferative hNP cells to post mitotic neurons continuously exposed to β-estradiol resulted in cytotoxic effects early on in the cultures that extended to disruption of neurite formation via a non-ER mechanism later in this continuum. Bis1 was more toxic to hNP cells than post mitotic neurons. BPA had no cytotoxic DNT effects and only at the highest dose affected neurite outgrowth parameters. Based on the data above which show neuron development endpoint specificity, our model could be efficient in large-scale drug and environmental neurotoxicity screening using informative hPSC-derived cultures.

## Conclusions

Compared to existing rat cortical neural culture and previously used acute hPSC-derived neural cell differentiation models, our model more accurately recapitulates chronic exposure to compounds that occur during developmental neurotoxicity. This DNT assay could help in screening of compounds throughout neurogenesis in the neural tube, the transition from neural progenitors through primarily post mitotic neurons. In summary, results here clearly suggest that the cumulative effects of known and potential neurotoxicants during neural maturation are significant at substantially lower levels than previously known. Taken together these data suggest this human DNT neurogenesis model is more sensitive than acute single cell type endpoint measures, and is a useful initial screening model for potential toxicants during early development of the CNS.

## Additional file

Additional file 1: Figure S1. Continuous Bis 1 exposure during differentiation for 14 DIV showed higher sensitivity compared to acute exposure. hNP cells were seeded onto 96 well plates at a density of 15,000 cells/well. Differentiating hNP cultures were fixed at end of DIV 14 for analysis following immunocytochemistry for βIII-tubulin and hoechst staining. hN2™ were seeded onto 96 well plates at a density of 15,000 cells/well and fixed by end of 24 h. A, B: 14 DIV differentiation and immunocytochemistry staining for hoechst and βIII-tubulin. A, non-treated DIV 14 cells; B, 10 μM Bis 1 treated DIV 14 cells. C, D: hN2™ 24 h incubation and immunocytochemistry staining for hoechst and βIII-tubulin. C, non-treated hN2™ cells; B, 10 μM Bis 1 treated hN2™ cells. E-H: Total cell count and neurite outgrowth quantification in control and 10 μM Bis 1 treatment in hN2™ cells. Scale bars = 50 μm. Values are the means ± SD. *significant difference between group (*P* < 0.05) (TIF 2790 kb).
