# Supplementary figures and images for: High content imaging quantification of multiple in vitro human neurogenesis events after neurotoxin exposure
Source: BMC Pharmacol Toxicol. 2016 Dec 1;17:62. doi: 10.1186/s40360-016-0107-4 (PMC5131404; doi:10.1186/s40360-016-0107-4)

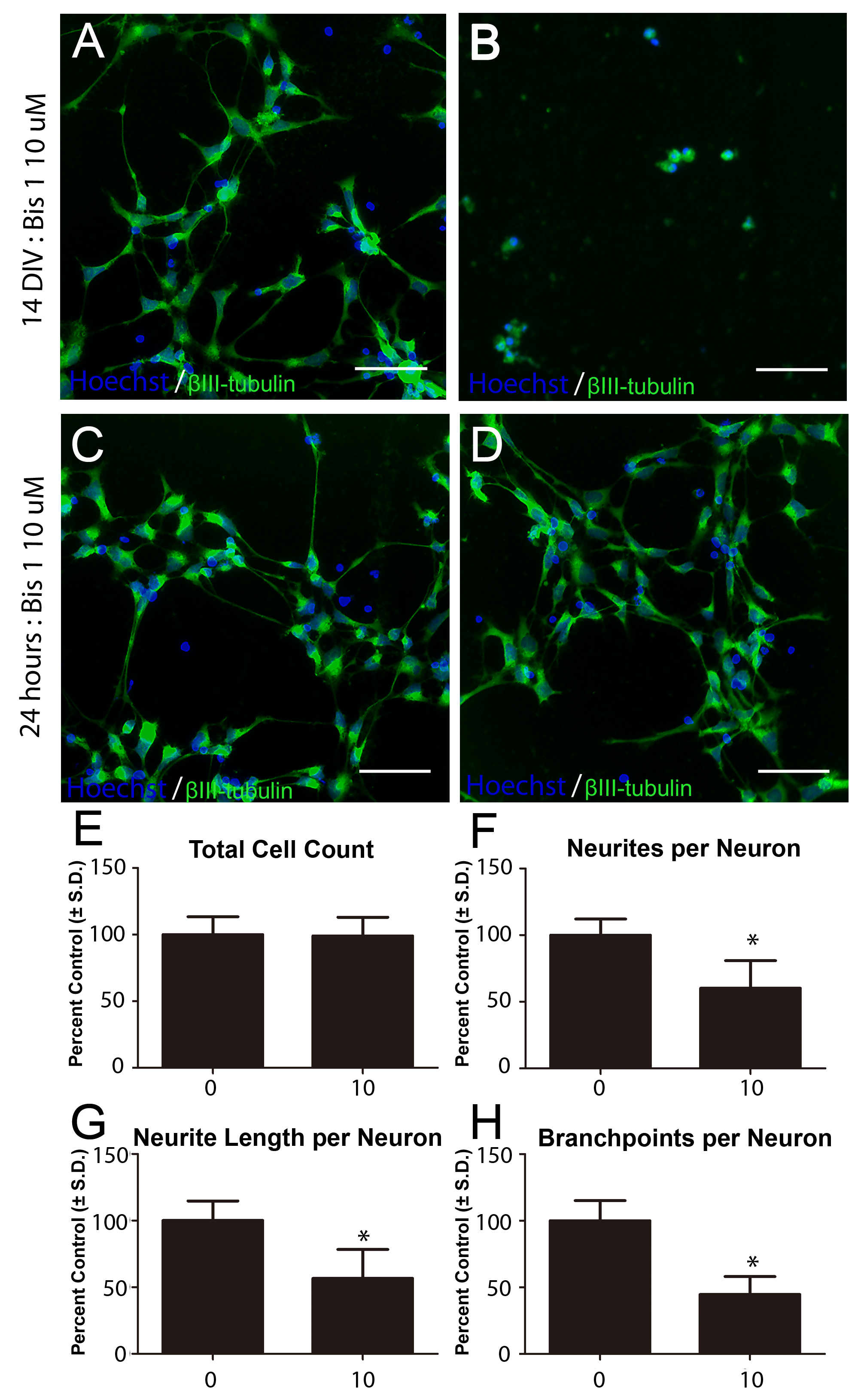

Supplement: Additional file 1: Figure S1. — Continuous Bis 1 exposure during differentiation for 14 DIV showed higher sensitivity compared to acute exposure. hNP cells were seeded onto 96 well plates at a density of 15,000 cells/well. Differentiating hNP cultures were fixed at end of DIV 14 for analysis following immunocytochemistry for βIII-tubulin and hoechst staining. hN2™ were seeded onto 96 well plates at a density of 15,000 cells/well and fixed by end of 24 h. A, B: 14 DIV differentiation and immunocytochemistry staining for hoechst and βIII-tubulin. A, non-treated DIV 14 cells; B, 10 μM Bis 1 treated DIV 14 cells. C, D: hN2™ 24 h incubation and immunocytochemistry staining for hoechst and βIII-tubulin. C, non-treated hN2™ cells; B, 10 μM Bis 1 treated hN2™ cells. E-H: Total cell count and neurite outgrowth quantification in control and 10 μM Bis 1 treatment in hN2™ cells. Scale bars = 50 μm. Values are the means ± SD. *significant difference between group (P < 0.05) (TIF 2790 kb). [file 40360_2016_107_MOESM1_ESM.tif]
